# Hypomethylation of *CNTFRα* is associated with proliferation and poor prognosis in lower grade gliomas

**DOI:** 10.1038/s41598-017-07124-9

**Published:** 2017-08-01

**Authors:** Kun Fan, Xiaowen Wang, Jingwen Zhang, Romela Irene Ramos, Haibo Zhang, Chunjie Li, Dan Ye, Jiansheng Kang, Diego M. Marzese, Dave S. B. Hoon, Wei Hua

**Affiliations:** 1 Institutes of Biomedical Sciences, Shanghai Medical College, Fudan University, Shanghai, China; 2 Department of Neurosurgery, Huashan Hospital, Fudan University, Shanghai, China; 3 Department of Translational Molecular Medicine, John Wayne Cancer Institute (JWCI), Providence Saint John Health Center, Santa Monica, CA United States of America; 4 Department of Ultrasound Diagnosis, Hebei General Hospital, Shijiazhuang, Hebei Province China; 5 Institute for Nutritional Sciences, Shanghai Institutes for Biological Sciences, Chinese Academy of Sciences, Shanghai, China; 6 Sequencing center, John Wayne Cancer Institute (JWCI), Providence Saint John Health Center, Santa Monica, CA United States of America

## Abstract

Ciliary neurotrophic factor receptor α subunit (CNTFRα) and CNTF play important roles in neuron survival, glial differentiation and brain tumor growth. However, the molecular mechanisms of *CNTFRα* regulation and its clinical significance in glioma remain largely unknown. Here, we found CNTFRα was overexpressed in lower grade gliomas (LGG) compared with glioblastoma (GBM) and normal brain specimens in TCGA datasets and in an independent cohort. Bioinformatics analysis revealed a CpG shore of the *CNTFRα* gene regulated its mRNA expression in TCGA datasets. This observation was further validated with clinical specimens and functionally verified using demethylating agents. Additionally, we observed that independent of *IDH* mutation status, methylation of *CNTFRα* was significantly correlated with down-regulated *CNTFRα* gene expression and longer LGG patient survival. Interestingly, combination of *CNTFRα* methylation and *IDH* mutation significantly (p < 0.05) improved the prognostic prediction in LGG patients. Furthermore, the role of CNTFRα in glioma proliferation and apoptosis through the PI3K/AKT pathways was demonstrated by supplementation with exogenous *CNTF  in vitro* and siRNA knockdown *in vivo*. Our study demonstrated that hypomethylation leading to *CNTFRα* up-regulation, together with autocrine expression of CNTF, was involved in glioma growth regulation. Importantly, DNA methylation of *CNTFRα* might serve as a potential epigenetic theranostic target for LGG patients.

## Introduction

Glioma is one of the most common primary brain tumors. Despite surgical resection and chemo-radiotherapy, the prognosis of glioblastoma (GBM) patients remains dismal. Lower grade gliomas (LGG) inevitably progress to high grade. A hallmark of GBM is the acquisition of self-sufficient growth factor signals^1^. Aberrant growth factors and receptors such as EGF/EGFR^2^, IGF/IGFR^3^, PDGF/PDGFR^4^, and CNTF/CNTFR^5^, become constantly active and contribute to glioma progression. Consequently, these growth factors have been the focus as molecular markers and therapeutic targets for gliomas. The activation of growth factor-related pathways such as *EGFR* has been well defined in glioma genesis, and could serve as diagnostic and therapeutic targets^6–8^.

Neurotrophic factors have specific roles in the brain. However, CNTF and its receptor CNTFRα have not been well demonstrated in gliomas. Upon binding to CNTF, CNTFRα recruits subunits gp130 and LIFRβ, and activates the downstream PI3K/AKT and MAPK/ERK pathways^5, 9^. CNTFRα plays multiple roles in promoting neuron proliferation, differentiation^10^, and protection^11^, and also is involved in liver cancer^12^. Previous studies showed that CNTFRα is a helpful marker for identifying tumor-initiating cells in gliomas and that CNTF might affect human glioma cells in both autocrine and paracrine fashion^13, 14^. However, the mechanism by which CNTF/CNTFRα is regulated remains largely unknown.

The objective of the present study was to investigate the role of CNTF/CNTFRα and its regulation in gliomas. To this end, the expression of *CNTFRα* in gliomas was analyzed in both the TCGA dataset and in an independent clinical sample collection. Thus, *CNTFRα* expression was compared between LGG, GBM, and normal brain specimens, and the role of DNA methylation in the regulation of *CNTFRα* expression was investigated. In addition, the effects of exogenous CNTF on glioma growth and knockdown of *CNTFRα* by siRNA on tumor inhibition were investigated both *in vivo* and *in vitro*. Our results demonstrated a relationship between the DNA methylation of a CpG island shore located in the first intron of *CNTFRα*, experimental tumor growth and poorer survival in LGG patients.

## Materials and Methods

### Antibodies and biochemical reagents

The following antibodies were used in this study: anti-CNTF Ab (Abcam Cambridge, MA, USA, Cat. # ab46172), anti-BAX Ab (Abcam Cambridge, MA, USA,Cat. # ab32502), anti-BCL2 Ab (Abcam Cambridge, MA, USA, Cat. # ab117115), anti-Ki67 Ab (Abcam, Cambridge, MA, USA, Cat. # 16667), anti-Wee1 Ab (Cell Signaling, Beverly, MA, USA, Cat. # 4938 S), anti-AKT Ab and anti-p(Ser473)-AKT Ab (Cell Signaling, Beverly, MA, USA, Cat. # 2920 S and 4060 P), anti-cleaved Caspase 3 Ab (Cell Signaling, Beverly, MA, USA,Cat. # 9664), anti-CNTFRα Ab (Santa Cruz, USA. Cat. # sc-9993), goat anti-mouse IgG Fluor488 Ab, goat anti-rabbit IgG Fluor 546 Ab (Invitrogen, NY, USA, Cat. # A11017 and A11071), anti-GAPDH Ab and mouse and rabbit secondary Ab (Kangcheng, Shanghai, China. Cat. # KC-5G4, KC-MM-035, KC-RB-035).

Other reagents and kits used were: CCK8 (Dojindo, Japan, Cat. # CK04), Annexin V-FITC cell apoptosis assay kit, Cell cycle assay kit (Beyotime, Shanghai, China, Cat. #C1063and C1052), LY294002 (Cell Signaling, Beverly, MA, USA, Cat. #9901), 5-Aza-2-dC (Sigma Aldrich, Saint Louis, MO, USA, Cat. #A3656), *CNTFRα* siRNA (Santa Cruz, USA, Cat. #35076), SYBR real time kit (Takara, Dalian, China, Cat. #DDR041A), MSP-PCR kit (Epigentek, Farmingdale, NY, USA, Cat. # P-1016-80).

### Cells and tissues

All glioma primary cell lines and tissues from Huashan Hospital (proved as WHO grade IV) were approved by Huashan Hospital Human Research Ethics Committee (ethics approval number KY2015-256). Informed consents had been obtained from all participants. SHG66 was isolated from fresh glioma tissue and established as a glioma cell line from Huashan Hospital. Human glioma cell lines U87, A172, LN18, U138, and U251 were obtained from the American Type Culture Collection. Cells were cultivated in Dulbecco’s modified Eagle’s medium (DMEM) (Invitrogen, NY, USA) supplemented with 10% fetal bovine serum at 37 °C with 5% CO_2_. Glioma frozen tissues and paraffin slides were obtained from Huashan Hosptital, Fudan University, Shanghai, China, with consent from every patient under the approved Human Research Ethics Committee protocol.

### Western blot

Tumor samples were prepared as follows. About 0.5 mg fresh tissues were weighed, cut up and washed with pre-chilled PBS, then lysed in RIPA buffer (50 mM Tris-HCl, pH 7.4, 150 mM NaCl, 1% NP-40, 0.5% sodium deoxycholate, 0.1% SDS, 1 mM EDTA) with protease inhibitor (1 mM Na_3_VO_4_, 1 mM PMSF), followed by centrifugation at 12,000 rpm for 20 minutes at 4 °C. Protein concentrations were assayed with the BCA protein concentration assay kit (KANGWEI, Shanghai, China, Cat. #CW0014S) according to the manufacturer’s instructions. Protein samples were separated by SDS-PAGE, transferred onto nitrocellulose membranes, blocked with 5% non-fat milk in TBST for 1 hour at room temperature, then incubated with specific primary antibodies overnight at 4 °C. The membranes were washed three times with TBST for 10 minutes, and then incubated with HRP-conjugated IgG secondary antibody for 2 hours at room temperature. The membranes were washed three times with TBST for 10 minutes. The protein was finally visualized by fluorography using an enhanced chemiluminescence system.

### Immunofluorescence staining

Cells were seeded on coverslips in a 24-well plate. When cell confluence reached 70–80%, the coverslip was washed with cold phosphate buffered saline (PBS), fixed with 4% paraformaldehyde for 30 minutes, permeabilized with 0.1% Triton X-100 for 10 minutes on ice, then blocked with 3% bovine serum albumin (BSA) for 1 hour at room temperature. The coverslips were incubated overnight at 4 °C with selected primary antibodies at 1:300-500 dilution in 3% BSA. After washing three times for 5 minutes with PBS, coverslips were incubated with goat anti-mouse Fluor488 or goat anti-rabbit Fluor 546 antibody at 1:500, then incubated with 2 µM DAPI for 1 minute, and then washed and sealed. The immunofluorescence stain was observed using a laser confocal microscope.

### Immunohistochemistry staining

Tumor xenografts were fixed in 4% paraformaldehyde then paraffin embedded and 0.5 μm sections were prepared. The process was performed as previously described^15^.

### siRNA interference and real-time PCR

Cells were seeded into 6-well culture dishes. When cell confluence reached 50–60%, cells were transfected with *CNTFRα* siRNA (Santa Cruz, USA, Cat. #sc-35076) using lipofectamine 2000 according to the manufacturer’s instructions for 48 hours before harvesting. Total RNA was isolated using TRIzol (Invitrogen, NY, USA, Cat. #15596026). Reverse transcription was performed according to the Promega reverse-transcription kit manufacturer’s instructions. About 2 μg RNA was used to reverse-transcribe each sample. In the first step, RNA was mixed with oligo (dT)18 and RNase-free water, then heated to 65 °C for 5 minutes, placed in an ice-bath for 5 minutes, then centrifuged at 12000 rpm for 2 minutes. The second step was to add reverse transcriptase and buffer to 20 μl at 37 °C for 1 hour, then 70 °C for 5 minutes, which completed reverse transcription. Real time PCR was performed with the SYBR Green kit to analyze *CNTFRα* mRNA level according to the manufacturer’s instructions. The reaction system was cDNA 4.5 μl (100 ng/μl), 2 × SYBR mix 5 μl, forward primer and reverse primer respectively 0.2 μl, ROX 0.1 μl, to a total volume of 10 μl. The relative expression levels were calculated according to the threshold cycle values of the samples. Real time PCR analysis was performed in triplicate for each sample in at least three independent experiments. The real-time PCR primers were: *CNTFRα* forward primer: GCCCGAGAAAGGACTCTAGC, *CNTFRα* reverse primer: ATGGCAGTGTCACGTCAGAG, *GAPDH* forward primer: CTCTGCTCCTCCTGTTCGAC, *GAPDH* reverse primer: TTCCCGTTCTCAGCCTTGAC.

### Cell viability, cell cycle, cell apoptosis analysis

Cell proliferation was assessed with the Cell Counting Kit-8 assay. Cells were transfected with *CNTFRα* siRNA for 24 h, and then plated into a 96-well dish. Cell viability was detected according to the manufacturer’s instructions at 0, 24, 48 and 72 h. Cells transfected with *CNTFRα* siRNA for 48 h were analyzed for cell cycle and apoptosis by flow cytometry according to the manufacturer’s instructions using the cell cycle and apoptosis assay kit (Beyotime, China).

### Methylation-specific PCR (MSP-PCR)

The MSP-PCR assays were performed according to the manufacturer’s instructions. DNA was extracted from paraffin-embedded tissue sections approximately 10 µm thick. Paraffin was removed using xylene then alcohol. The DNA sample was then extracted using the TiANamp FFPE DNA Kit (TIANGEN, Beijing, China, Cat. #DP330) according to the manufacturer’s recommendations. Sodium bisulfite modification of DNA was performed according to the manufacturer’s instructions using the Methylamp Whole Cell Bisulfite Modification Kit. Briefly, DNA was denatured in 0.3 M NaOH at 37 °C for 15 min, then 10 mM hydroquinone and 3.6 M sodium bisulfite was added to treat the DNA sample in a 55 °C water bath for 16 h. This converts cytosine residues to uracil in single-stranded DNA while leaving methylated cytosine unchanged. These DNA samples were desalted and desulfonated, then precipitated with ethanol and dissolved in double distilled water. The methylation status of the *CNTFRα* promoter was assessed by MSP-PCR using two sets of primers, methylated (M) or unmethylated (U) primers. The primers were: *CNTFRα*-S-M/U: ACCTCTATCTCTCCATATCAAAC, *CNTFRα*-AS-M: 5′-CTATAATCACTAAAATAATACG-3′ and *CNTFRα*-AS-U: 5′-CTATAATCACTAAAATAATACA. In brief, quantitative MSP-PCR was performed as follows. 1 μg of DNA standard was processed as described above to obtain treated DNA. Standard DNA was isolated by concentration gradient and the sample was subjected PCR. PCR products were separated by agarose gel electrophoresis and quantified by density analysis.

### Tumor xenograft growth assay *in vivo*

With the approval of the Ethics Committee of Fudan University, animal experiments were carried out in accordance with the Guide for the Care and Use of Laboratory Animals. Nude mice were purchased from SLAC Laboratory Animal Corp (Shanghai, China). Firstly, A172 and U87 glioma cells (1 × 10^6^) were inoculated sub-dermally into 5–6 week old female BALB/c nude mice on the abdomen. Tumor xenograft growth was monitored every other day. When the diameter of the tumor reached about 1 cm, at around day 19, the tumor-bearing mice were divided randomly into two groups to ensure equal tumor xenograft volumes. In the experimental group, 5 nmol siRNA targeting *CNTFRα* dissolved in 100 ul PBS (pH 7.4) was rapidly injected into tumor xenograft every other day (from day 19). The control group was injected by the same schedule with non-targeting siRNA. Animals were monitored every day. Xenograft tumors were harvested and photographed on the twenty-ninth day. Tumor xenograft dimensions were measured with calipers and the volume (mm^3^) calculated as width^2^ × length/2. Tumor weight was measured as well. Tumor tissues were processed for western blot and immunohistochemical assay.

### Bioinformatics and statistical analysis

LGG and GBM TCGA datasets were obtained from the latest version (July 15, 2016) (http://cancergenome.nih.gov/). mRNA expression, clinical data, and DNA methylation were extracted using the “data matrix” tool of the TCGA data portal. DNA methylation data from the Illumina Infinium Human DNA Methylation 450 and mRNA expression data from Illumina HiSeq. 2000 RNA Sequencing version 2 was integrated for the analysis. The correlation between DNA methylation level and gene expression was evaluated, as previously described^15^. For DNA methylation, the cutoff for hyper- and hypo-methylation of *CNTFRα* was selected using the median beta value (m = 0.376, n = 530). The statistical differences between DNA methylation and gene expression levels were assessed by Student’s t test or Wilcoxon’s Rank test according to the distribution of the variable. The results were presented as mean ± SD of samples measured in technical and biological triplicates. Correlation analysis was performed using Pearson’s R correlation coefficient. Survival analysis was conducted using the Kaplan–Meier method and comparisons among groups were performed using log-rank tests in JMP9.0 (SAS Institute Inc., Cary, NC). Differences were considered significant at p < 0.05 for all the statistical analysis.

## Results

### CNTFRα was overexpressed in lower grade gliomas

**Figure 1:**
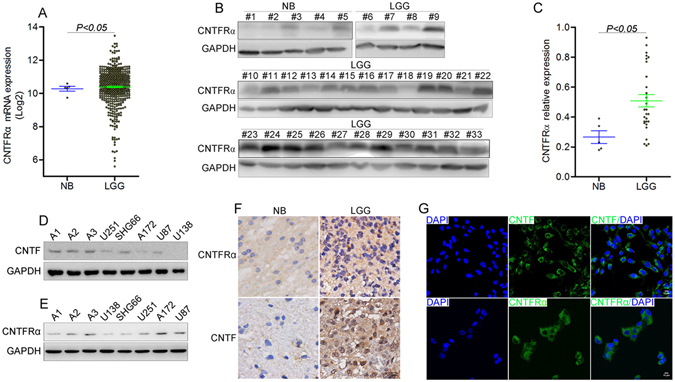
CNTFRα was overexpressed in LGG and CNTF can act as an autocrine ligand in glioma. (**A**) Bioinformatic analysis of *CNTFRα* expression in LGG (n = 530) and normal brain tissues (NB, n = 5, p < 0.05). (**B**) CNTFRα protein expression was analyzed in glioma samples (LGG n = 28) with normal brain tissue as control (n = 5). (**C**) Densitometric analysis of CNTFRα protein expression level of glioma samples. (**D**) and (**E**) Western blot results of CNTF and CNTFRα protein expression in three glioma primary cells and cell lines. (**F**) Immunofluorescence analysis of CNTF and CNTFRα expression showed membrane location (﻿scale bar 10 um). (**G**) IHC analysis of CNTF and CNTFRα expression in glioma tissues. Western blots were representative of three independent experiments. Statistical results represent the mean ± SD of n = 3 determinations.

Bioinformatics analysis of TCGA glioma datasets revealed that *CNTFRα* mRNA level was significantly higher in LGG (n = 530) than that in normal brain tissues (n = 5) (Fig. 1A, p < 0.05, fold change = 0.42). Compared with GBM (n = 167), *CNTFRα* mRNA expression was significantly higher in LGG (n = 530) (Fig. S1A, p < 0.01, fold change = 0.98), In GBM molecular subgroup analysis, the proneural subgroup had higher *CNTFRα* expression than other groups (p < 0.001, n = 202, Fig. S1B). The overexpression of CNTFRα in LGG (n = 28) was further confirmed at the protein level by western blot analysis of our clinical samples compared with normal brain tissues (n = 5, p < 0.05, Fig. 1B and C). IHC staining also demonstrated that CNTFRα expression was higher in LGG than normal brain tissue (Fig. 1F). Besides, *CNTF* mRNA expression was also significantly higher in LGG (n = 530) than GBM (n = 167) (Fig. S1C, p < 0.01, Fold change = 0.63). CNTFRα and CNTF expression were profiled in three primary glioma cells and five established cell lines. CNTFRα was overexpressed in most cell lines (Fig. 1D and E). Immunofluorescence staining showed that CNTFRα was mainly located on the cell membrane, while CNTF was cytoplasmic (Fig. 1G). Together, the data indicated that CNTFRα was overexpressed in LGG.

### Methylation of a CpG Island shore in *CNTFRα* regulated *CNTFRα* mRNA expression

**Figure 2:**
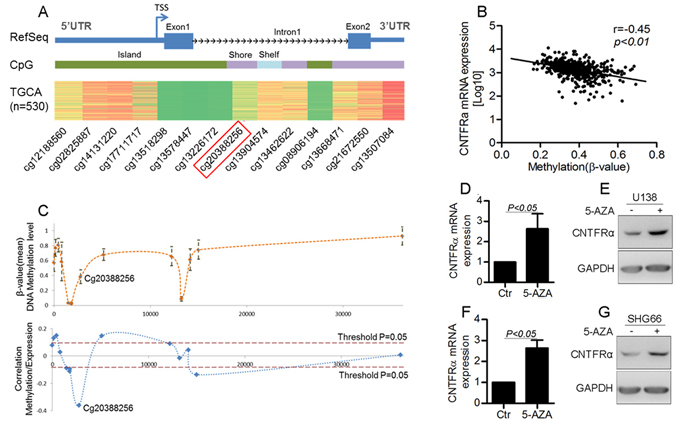
Methylation of a CpG island shore in the *CNTFRα* gene regulated *CNTFRα* mRNA expression. (**A**) *CNTFRα* gene structure [based on RefSeq Feb. 2009 (GRCh37/hg19) assembly], and heat map showing DNA methylation level throughout the *CNTFRα* gene from the TCGA LGG cohort (n = 530). Blue boxes represent the first and second exons. Green, purple and aqua represent the CpG context (CpG island, CpG shore and CpG shelf), respectively. DNA methylation level throughout *CNTFRα* gene in LGG. (**B**) Scatter plot of the correlation analysis between CNTFRα mRNA and methylation level of probe cg20388256 (r = −0.45, p < 0.0001). (**C**) Correlation analysis between DNA methylation and gene expression levels. Each point represented one CpG site and the dashed line indicateed the variation of correlation throughout *CNTFRα* gene. Orange lines represented the statistically significant threshold for the correlation analysis (p = 0.05). (**D**,**E**,**F** and **G**) U138 and SH66 cells were treated with 5-Aza-2-dC or control, and *CNTFRα* expression was quantified by real time PCR and Western blot. Error bars represented means ± SD from replicates (n = 3).

Analysis of the data from RNAseq and methylation array in the TCGA LGG dataset revealed a significant inverse correlation between *CNTFRα* mRNA expression and the methylation level of 5 (1939, 1706, 757, 751, 427 bps from TSS) out of 14 potential methylation sites around the *CNTFRα* promoter (p < 0.05, Fig. 2A and C). The CpG island shore of the *CNTFRα* in the first intron (cg20388256, 1706 bps from the TSS) was the most significant one related with mRNA expression (Spearman correlation, r = −0.45, p < 0.0001, Fig. 2B). These results were consistent with the analysis of the TCGA GBM dataset (Fig. S2A,B and C), which, too, indicated that methylation of the CpG island shore in the the first intron of *CNTFRα* gene contributed to the regulation of *CNTFRα* expression.

**Figure 3:**
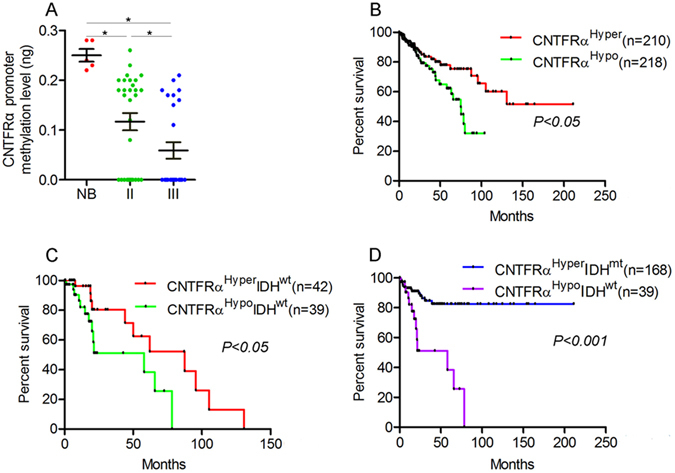
*CNTFRα* hypomethylation could predict poor survival in LGG. (**A**) The methylation level of the cg20388256 probe region was quantitatively analyzed by MSP -PCR. LGG (n = 62) had significant lower methylation level than normal brain tissues (n = 6, p < 0.05). (**B**) *CNTFRα* ^hyper^ group was associated with longer patient survival compared with *CNTFRα* ^hypo^ group (p < 0.05). (**C**) *CNTFRα* ^hyper^ group was associated with longer survival compared with *CNTFRα* ^hypo^ in *IDH* ^wt^ patients. (**D**) *CNTFRα* ^hyper^ *IDH* ^mt^ group was associated with longer survival compared with *CNTFRα* ^hypo^ *IDH* ^wt^ (p < 0.05). hyper: hypermethylation; hypo: hypomethylation; mt: mutation; mt: wide type.

To verify the regulation of *CNTFRα* expression by methylation, U138 and SHG66 glioma cells with low expression level of *CNTFRα* (Fig. 1E) were treated with different doses of 5-Aza-2dC^16^. The *CNTFRα* mRNA and protein expression were significantly increased (Fig. 2D–G, S2F and G, p < 0.05) together with lower methylation level (Fig. S2D and E, p < 0.05). Further analysis showed that *CNTFRα* mRNA expression had significant inverse correlation with CpG island shore methylation of *CNTFRα* (Fig. S2H and I, Spearman correlation, r = −0.742 and −0.892, p < 0.0001).The assay of clinical samples further verified that *CNTFRα* methylation level was significantly decreased in LGG compared with normal brain tissues (Fig. 3A).

### *CNTFRα* hypomethylation was associated with poor patient survival in LGG

The clinical significance of *CNTFRα* hypomethylation was investigated. The methylation of the CpG island shore site was analyzed by MSP-PCR in clinical samples and normal brain tissues. The MSP-PCR results showed a lower methylation level in glioma tissues compared with normal brain tissues (LGG, n = 63, normal brain tissue, n = 6, p < 0.05, Fig. 3A and S3A). The prognostic value of this marker was investigated in the TCGA LGG glioma dataset. Patients with hypomethylated *CNTFRα* showed poorer survival than those with hypermethylated *CNTFRα* in LGG cohort (p < 0.05, Fig. 3B). *IDH* mutation is an independent prognostic marker^17^, so we incorporated this into our analysis. There was no significant difference either in methylation level (p = 0.754) or mRNA expression level (p = 0.651) of *CNTFRα* between different *IDH* mutation status in LGG (Fig. S4A and B). However, when the two factors were combined, *CNTFRα* ^hypo^ (hypomethylation of *CNTFRα*) in *IDH* ^wt^ (*IDH* wild type) patients was significantly correlated with poorer survival compared with *CNTFRα* ^hyper^ (hypermethylation of *CNTFRα*) patients (p < 0.05, Fig. 3C). Additionally, *CNTFRα* ^hypo^ *IDH* ^wt^ patients had much worse survival than *CNTFRα* ^hyper^ *IDH* ^mt^ (*IDH* mutant) patients (n = 56, p < 0.0001, Fig. 3D). The combination of two factors, *CNTFRα* ^hypo^ *IDH* ^wt^ and *CNTFRα* ^hyper^ *IDH* ^mt^ could provide a better assessment of LGG survival compared with either *CNTFRα* methylation or *IDH* mutation alone (p < 0.01, Fig. S4C and D). In summary, hypomethylation of *CNTFRα* was correlated with poorer overall survival in LGG, and the combination of *IDH* mutation and *CNTFRα* methylation seems to better predict survival in LGG.

### CNTF/CNTFRα promoted glioma cell proliferation and inhibited apoptosis by the PI3K/AKT pathway *in vitro*

**Figure 4:**
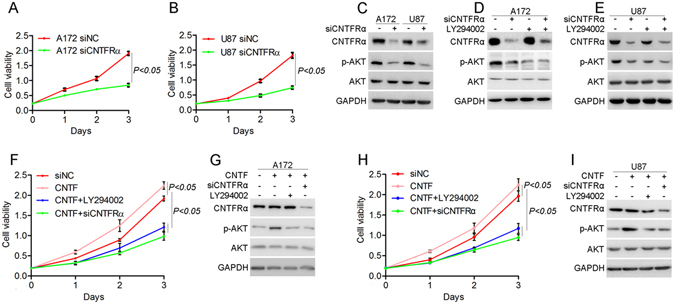
CNTF/CNTFRα promoted glioma cell proliferation by PI3K/AKT pathway *in vitro*. (**A**) A172 and (**B**) U87 cells were seeded into 96 well plate after transfection with siRNA. Cell viability was assayed by CCK8 at indicated time points. (**C**,**D** and **E**) p-AKT was analyzed by western blot after transfecting siRNA or LY294002 treatment. (**F**) and (**H**) A172 and U87 cell viability was assayed with CNTF, LY294002 or siRNA treatment. (**G**) and (**I**) p-AKT was analyzed by Western blot after transfection with siRNA or LY294002 treatment.

To explore the function of CNTF/CNTFRα pathway in glioma, *CNTFRα* siRNA and exogenous CNTF were used to treat glioma cell lines. A172 and U87cells, with higher CNTFRα expression (Fig. 1E) were transfected with *CNTFRα* siRNA. The cell viability was significantly decreased (p < 0.01 Fig. 4A and B). In parallel, cell count also significantly decreased after *CNTFRα* knockdown in A172 and U87cells (Fig. S5A and B). Pathway analysis showed that *CNTFRα* knockdown decreased the protein level of phosphorylated AKT (p-AKT S473) in A172 and U87 (Fig. 4C), showing that *CNTFRα* knockdown could inhibit the PI3K/AKT pathway. A well-characterized inhibitor of PI3K, LY294002, was employed as a control of the inhibitor, either alone or in combination with *CNTRFα* siRNA. Both *CNTFRα* knockdown and LY294002 treatment decreased p-AKT protein levels to about the same extent, and combination treatment down-regulated the p-*AKT* level only slightly compared with single treatment. These results identified the PI3K/AKT pathway as the downstream signaling pathway of CNTFRα (Fig. 4D and E).

Exogenous CNTF treatment significantly increased cell viability in A172 cells. Both LY294002 treatment and *CNTFRα* knockdown overcame the increased cell viability induced by CNTF (Fig. 4F and H). Similarly, CNTF treatment activated the PI3K/AKT pathway by elevating p-AKT expression level. Both LY294002 treatment and *CNTFRα* knockdown negated the effect of exogenous CNTF, leading to pathway inactivation (Fig. 4G and I). Taken together, this evidence strongly suggested that CNTF/CNTFRα could promote cell proliferation through the activation of PI3K/AKT pathway *in vitro*.

**Figure 5:**
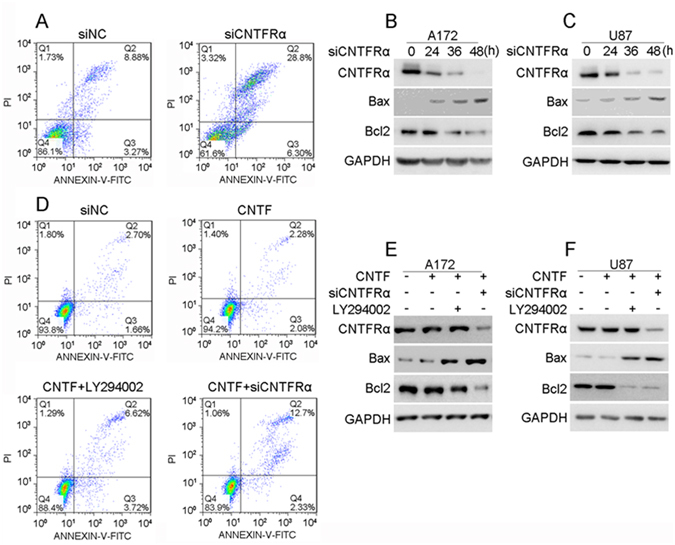
CNTF/CNTFRα inhibited apoptosis by PI3K/AKT pathway *in vitro*. (**A**) U87 and (**D**) A172 cell apoptosis were analyzed with Annexin V and Propidium Iodide by flow cytometry assay after CNTF, si*CNTFRα* or LY294002 treatment. (**B**,**C**,**E**,**F**) Apoptosis- related protein BCL2 and BAX were detected by western blot after siRNA transfection, CNTF and LY294002 treatment in U87 and A172 cells. western blot was representative of three independent experiments.

Knockdown of *CNTFRα* significantly increased cell apoptosis. In A172 and U87 cells, *CNTFRα* siRNA significantly induced more apoptosis compared with controls (17.76% *vs* 6.16%, p < 0.05; 35.1% *vs* 12.15%, p < 0.05) (Fig. 5A and D). The expression of BCL2 was gradually decreased with time after *CNTFRα* knockdown, while BAX expression increased, reflecting the process of apoptosis over a period of 48 h (Fig. 5B and C). Exogenous CNTF treatment did not induce significant apoptosis-resistance, and did not prevent the increase in apoptosis caused by LY294002 or *CNTFRα* knockdown even though supplemented with CNTF (Fig. 5E and F). However, *CNTFRα* knockdown had no influence on the cell cycle (Fig. S5C and E), which was confirmed by unchanged expression of cell cycle-related proteins including Wee1 and Cyclin D1 (Fig. S5D and F). These results were consistent with the hypothesis that *CNTFRα* knockdown significantly inhibited glioma cell proliferation and induced apoptosis by suppressing the PI3K/AKT pathway *in vitro*.

Besides the proliferation, differentiation markers such as GFAP had an inverse correlation with CNTFRα expression (Spearman correlation, r = −0.3, p < 0.01, Fig. S6A and B). Furthermore, GFAP expression was decreased after CNTF treatment in A172 detected by flow cytometry (Fig. S6C and D). However, there is no significant correlation between CNTFRα expression and other glioma differentiation markers in the TCGA LGG dataset (n = 534, Fig. S6E).

### Knockdown of *CNTFRα* inhibited glioma xenograft growth *in vivo*

**Figure 6:**
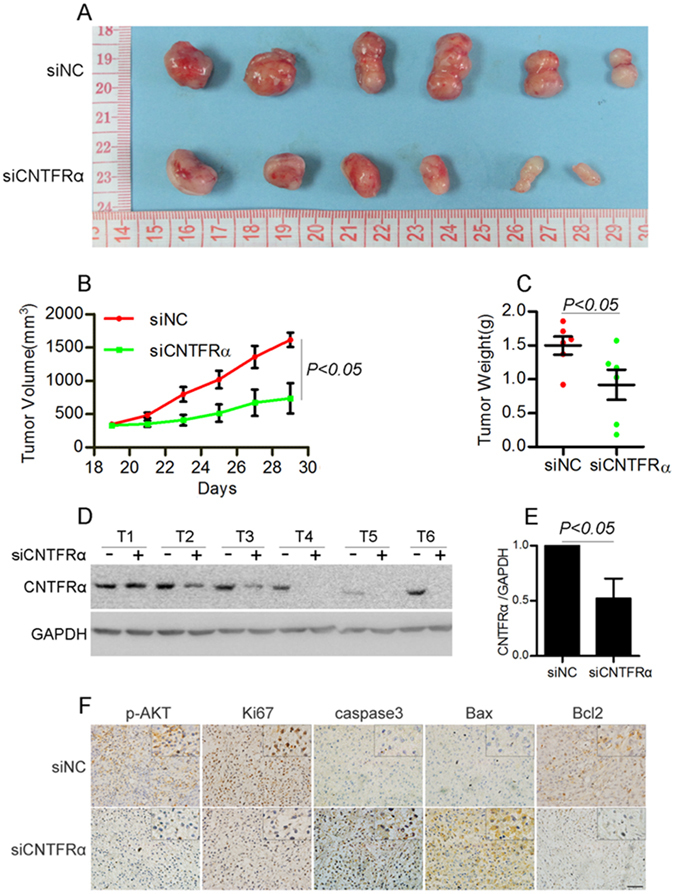
Knockdown of *CNTFRα* inhibited glioma xenograft growth *in vivo*. (**A**) A172 cells were injected subcutaneously into the addominal flanks of nude mice. When xenograft tumor volume was even on the nineteenth day, siRNA was injected into tumor xenografts every other day. Finally, xenograft tumors were harvested and photographed on the twenty-ninth day. (**B**) Xenograft tumor volume was decreased by 54% and (**C**) Xenograft tumor weight was decreased by 39% in the si*CNTFRα* group compared with control group. (**D**) and (**E**) In xenograft tumors, CNTFRα expression level was significantly decreased in the si*CNTFRα* group compared with the control group. Western blot results were representative of three independent experiments. (**F**) Immunohistochemistry analysis of the xenograft tumors showing decreased expression of p-AKT, BCL2, Ki67 and increased expression of cleaved caspase 3 and BAX in the si*CNTFRα* injection group.

To investigate the anti-glioma effect by inhibiting *CNTFRα in vivo*, A172 and U87 cells were subcutaneously seeded to nude mice. Tumor xenografts were successfully generated (Fig. 6A and S7A). CNTFRα expression was reduced in tumor xenografts injected with *CNTFRα* siRNA (Fig. 6D and E). The growth and size of the tumors were assessed. The tumor xenografts grew slower when CNTFRα was knocked down by siRNA. Tumor volume and weight were decreased about 54% and 39%, respectively, in the *CNTFRα* siRNA injection group compared with the control group (Fig. 6B and C, p < 0.05). The results were consistent in both A172 and U87 cells (Fig. S7B and C). Immunohistochemistry analysise was used to further identify the function of CNTFRα in vivo. Treatment of tumors with *CNTFRα* siRNA decreased the expression of p-AKT, BCL2 and Ki67, and increased the expression of cleaved Caspase 3 and BAX as observed in *CNTRFα* siRNA-transfected glioma *in vitro* (Fig. 6F). Here, we have demonstrated *CNTFRα* knockdown led to a significant inhibition of glioma growth *in vivo* by inhibiting proliferation and promoting apoptosis.

## Discussion

In this era of “multi-omics”, many new mechanisms and biomarkers have been discovered in gliomas, paving the way for “molecular pathology”^18^. CNTFRα expression has been identified as a helpful marker of tumor-initiating cells in gliomas^14^. However, the regulation of *CNTFRα* remains to be explored. In this study, it was demonstrated that methylation of CpG island shore of the *CNTFRα* gene could be a potential prognostic marker in LGG patients, Furthermore, CNTFRα, together with autocrine CNTF ligand, plays a tumor oncogene role by regulating the PI3K/AKT pathway.

Overexpression of CNTFRα has been found in many cancers, such as anaplastic large-cell lymphoma and hepatocellular carcinoma^12^. In our study, CNTFRα was up-regulated in LGG and GBM compared with normal brain tissues, although higher expression levels of CNTF and CNTFRα were observed in LGG compared with GBM. In the proneuronal subgroup of GBM, gene expression and methylation-based subtypes are relevant for LGG^19^. These data indicated that the overexpression of CNTFRα might play a more important role in LGG than in GBM.

Many members of growth factor receptors are known to be regulated by methylation in various diseases^20–22^. In this study, the correlation between CNTFRα expression and DNA methylation was investigated in the TCGA LGG dataset, assessed by MSP-PCR in clinical samples, and then verified in cell lines with 5-Aza-2dC. The CpG island shore (cg20388256, 1706 bps from the TSS) was identified as the most significant site regulated by methylation. In glioma, methylation is an important epigenetic regulation factor in tumor initiation, progression and prognosis^23, 24^. Here we showed that methylation not only regulated the *CNTFRα* mRNA expression, but also correlated with survival. Patients with hypermethylation of *CNTFRα* had better survival than the hypomethylation group in LGG with both *IDH* wild type and mutation status. It is known that *IDH* mutation patients, along with the glioma CpG island methylation phenotype (G-CIMP) of the whole genome, have a better prognosis than *IDH* wild type. *IDH* mutation and hypermethylation of *CNTFRα* were both good prognostic factors. The combination of *IDH* mutation and *CNTFRα* gene methylation provided a better prediction of survival. Although the mRNA of *CNTFRα* alone was not strong enough to be a potential prognostic marker due to the complexity and diversity of mRNA and protein expression, it could be considered as a strong candidate for prognosis when used in combination with multiple markers. Overall, this indicated methylation of the *CNTFRα* was a potential valuable prognostic marker in LGG patients.

Downstream signaling pathway of the CTNFRα had been well characterized in several studies. These studies demonstrated that PI3K/AKT, JAK2/STAT3 and MAPK/ERK signaling pathways mediate the response of neurons to cytokines^25–27^. CNTFRα acts as a modulator to regulate the crosstalk between the activation of PI3K and AMPK pathways, which contributes to CNTF-induced hepatic cancer growth^12^. In this study, *CNTFRα* knockdown in glioma cell lines decreased the p-AKT S473 level, leading to the inhibition of the PI3K/AKT pathway. On the contrary, exogenous CNTF activated the pathway. No significant change in the JAK2/STAT3 and MAPK/ERK signaling pathways was observed during this event (data not shown). Evidence from xenograft tumors also verified that down-regulated CNTFRα could modulate tumor growth by inhibiting proliferation and promoting apoptosis through the PI3K/AKT pathway. Recent studies suggested activation of the PI3K/AKT/mTOR pathway occurred in most adult LGG and was predictive of patient survival^28^. In our study, methylation of *CNTFRα* and autocrine CNTF from glioma cells activated the PI3K/AKT pathway, which could promote LGG progression.

This study has some limitations. GFAP expression was decreased after CNTF treatment, so CNTFRα may ﻿be﻿ involved with glioma stem cell differentiation, which was consistent with a previous report^14^. This indicated that CNTFRα may play multiple roles in gliomas. Downstream of CNTFRα functional study had shown various results between different cell lines. In Ozog MA.*et al*. study^8^, C6 rat cell line which lacks CNTFRα was used to explore the activation mechanism. Interestingly, we found inconsistent results with their study studies by using human GBM cell lines with CNTFRα high expression and TCGA database as well as clinical tissues. The mechanism as assessed in GBM cell lines may not be the best model for the LGG tumors *in vivo*, further studies are needed to examine CNTFRα in various pathological stages of glioma.

In conclusion, our study has shown that hypomethylation of a CpG island shore in the first intron of the *CNTFRα* gene is associated with LGG progression. Methylation of this CpG island shore may be a potential epigenetic theranostic marker for LGG. The application of this *CNTFRα* marker may benefit the precision treatment of LGG in the future.

## Electronic supplementary material

Supplementary Figures

Supplementary Figure legends

## References

[1] Kruttgen A, Schneider I, Weis J (2006). The Dark Side of the Ngf Family: Neurotrophins in Neoplasias. Brain Pathol..

[2] Tsuchihashi K (2016). The Egf Receptor Promotes the Malignant Potential of Glioma by Regulating Amino Acid Transport System Xc(-). Cancer Res..

[3] Maris C (2015). Igf-Ir: A New Prognostic Biomarker for Human Glioblastoma. Br J Cancer..

[4] Lu F (2016). Olig2-Dependent Reciprocal Shift in Pdgf and Egf Receptor Signaling Regulates Tumor Phenotype and Mitotic Growth in Malignant Glioma. Cancer Cell..

[5] Weis J (1999). Cntf and its Receptor Subunits in Human Gliomas. J Neurooncol..

[6] Hughes SM, Lillien LE, Raff MC, Rohrer H, Sendtner M (1988). Ciliary Neurotrophic Factor Induces Type-2 Astrocyte Differentiation in Culture. Nature..

[7] Park DM (2007). N-Cor Pathway Targeting Induces Glioblastoma Derived Cancer Stem Cell Differentiation. Cell Cycle..

[8] Ozog MA, Bechberger JF, Naus CC (2002). Ciliary Neurotrophic Factor (Cntf) in Combination with its Soluble Receptor (Cntfralpha) Increases Connexin43 Expression and Suppresses Growth of C6 Glioma Cells. Cancer Res..

[9] Ozog MA (2004). The Complex of Ciliary Neurotrophic Factor-Ciliary Neurotrophic Factor Receptor Alpha Up-Regulates Connexin43 and Intercellular Coupling in Astrocytes Via the Janus Tyrosine Kinase/Signal Transducer and Activator of Transcription Pathway. Mol Biol Cell..

[10] Linker RA (2002). Cntf is a Major Protective Factor in Demyelinating Cns Disease: A Neurotrophic Cytokine as Modulator in Neuroinflammation. Nat Med..

[11] Hashimoto Y, Kurita M, Aiso S, Nishimoto I, Matsuoka M (2009). Humanin Inhibits Neuronal Cell Death by Interacting with a Cytokine Receptor Complex Or Complexes Involving Cntf Receptor Alpha/Wsx-1/Gp130. Mol Biol Cell..

[12] Hu X (2008). Ciliary Neurotrophic Factor Receptor Alpha Subunit-Modulated Multiple Downstream Signaling Pathways in Hepatic Cancer Cell Lines and their Biological Implications. Hepatology..

[13] Lesser SS, Holmes TM, Pittman AJ, Lo DC (1999). Induction of Electrical Excitability by Ngf Requires Autocrine Action of a Cntf-Like Factor. Mol Cell Neurosci..

[14] Lu J (2012). Cntf Receptor Subunit Alpha as a Marker for Glioma Tumor-Initiating Cells and Tumor Grade: Laboratory Investigation. J Neurosurg..

[15] Marzese DM (2014). Epigenome-Wide Dna Methylation Landscape of Melanoma Progression to Brain Metastasis Reveals Aberrations On Homeobox D Cluster Associated with Prognosis. Hum Mol Genet..

[16] Derissen EJ, Beijnen JH, Schellens JH (2013). Concise Drug Review: Azacitidine and Decitabine. Oncologist..

[17] Louis DN (2016). The 2016 World Health Organization Classification of Tumors of the Central Nervous System: A Summary. Acta Neuropathol..

[18] Buckner J (2017). Management of Diffuse Low-Grade Gliomas in Adults - Use of Molecular Diagnostics. Nat Rev Neurol..

[19] Guan X (2014). Molecular Subtypes of Glioblastoma are Relevant to Lower Grade Glioma. PLOS One..

[20] Golzenleuchter M (2015). Plasticity of Dna Methylation in a Nerve Injury Model of Pain. Epigenetics-US..

[21] Li J, Jia XF, Liu J, Liu JJ, Zhao HB (2015). Relationship of Egfr Dna Methylation with the Severity of Non-Small Cell Lung Cancer. Genet Mol Res..

[22] Pan ZY, Jiang ZS, Ouyang HQ (2015). Study of the Methylation Patterns of the Egfr Gene Promoter in Non-Small Cell Lung Cancer. Genet Mol Res..

[23] Ceccarelli M (2016). Molecular Profiling Reveals Biologically Discrete Subsets and Pathways of Progression in Diffuse Glioma. Cell.

[24] Brennan CW (2013). The Somatic Genomic Landscape of Glioblastoma. Cell..

[25] Alonzi T (2001). Role of Stat3 and Pi 3-Kinase/Akt in Mediating the Survival Actions of Cytokines On Sensory Neurons. Mol Cell Neurosci..

[26] Sango K, Yanagisawa H, Komuta Y, Si Y, Kawano H (2008). Neuroprotective Properties of Ciliary Neurotrophic Factor for Cultured Adult Rat Dorsal Root Ganglion Neurons. Histochem Cell Biol..

[27] Liu H, Liu G, Bi Y (2014). Cntf Regulates Neurite Outgrowth and Neuronal Migration through Jak2/Stat3 and Pi3K/Akt Signaling Pathways of Drg Explants with Gp120-Induced Neurotoxicity *in Vitro*. Neurosci Lett..

[28] McBride SM (2010). Activation of Pi3K/Mtor Pathway Occurs in Most Adult Low-Grade Gliomas and Predicts Patient Survival. J Neurooncol..

