# Supplementary material for: Hypomethylation of CNTFRα is associated with proliferation and poor prognosis in lower grade gliomas
Source: Sci Rep. 2017 Aug 1;7:7079. doi: 10.1038/s41598-017-07124-9 (PMC5539284; doi:10.1038/s41598-017-07124-9)
Supplement: Supplementary file 1 — Supplementary Figures [file 41598_2017_7124_MOESM1_ESM.pdf]

Supplementary Figures

Hypomethylation of *CNTFRα* is associated with proliferation and poor prognosis in lower grade gliomas

Kun Fan[1,2*], Xiaowen Wang[1,3*], Jingwen Zhang[4*], Romela Irene Ramos[3], Haibo Zhang[1], Chunjie Li[1,2], Dan Ye[2], Jiansheng Kang[5], Diego M. Marzese[3], Dave S.B. Hoon[3,6], Wei Hua[#1,3]

Figure S1

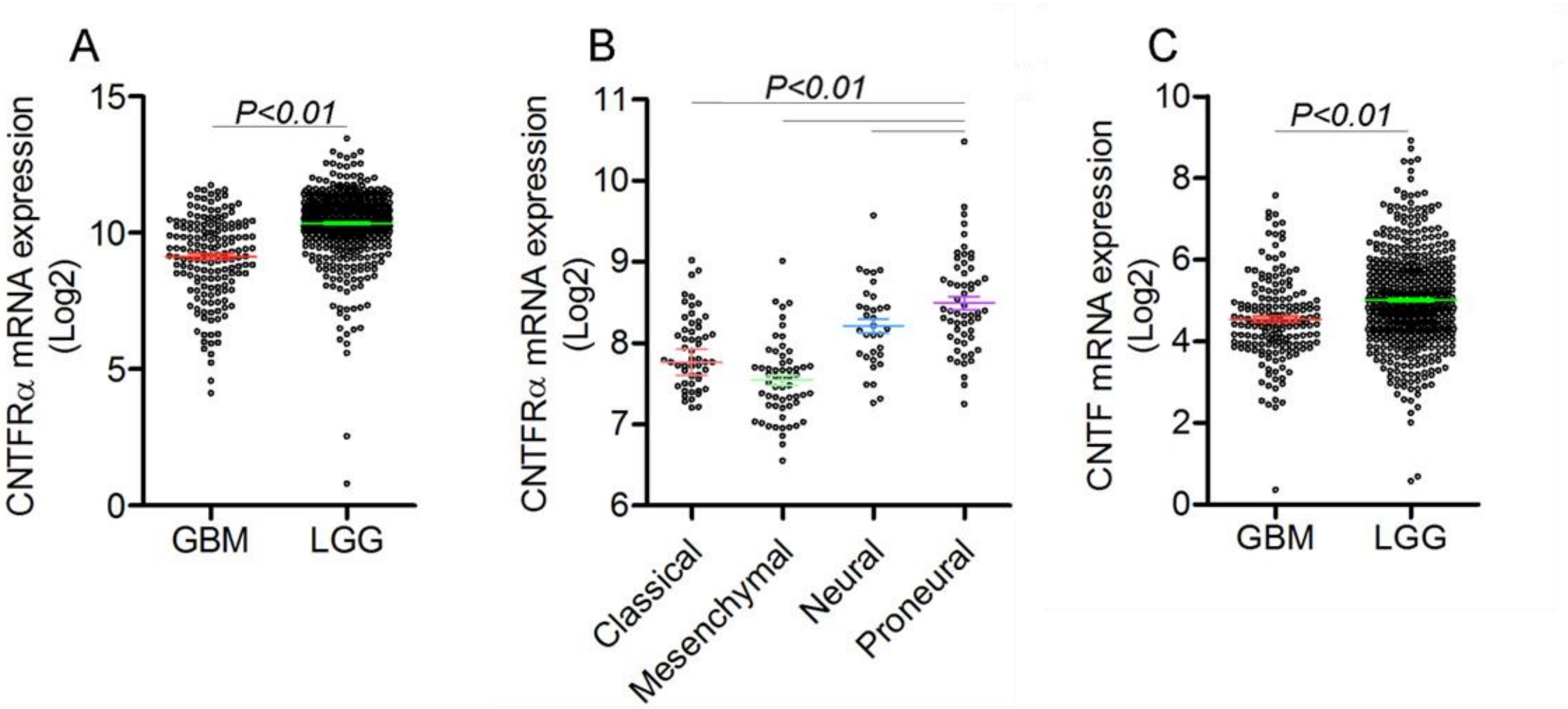
A
CNTFRα mRNA expression (Log2)
P<0.01
15
10
5
0
GBM
LGG
B
CNTFRα mRNA expression (Log2)
P<0.01
11
10
9
8
7
6
Classical
Mesenchymal
Neural
Proneural
C
CNTF mRNA expression (Log2)
P<0.01
10
8
6
4
2
0
GBM
LGG

Figure S2

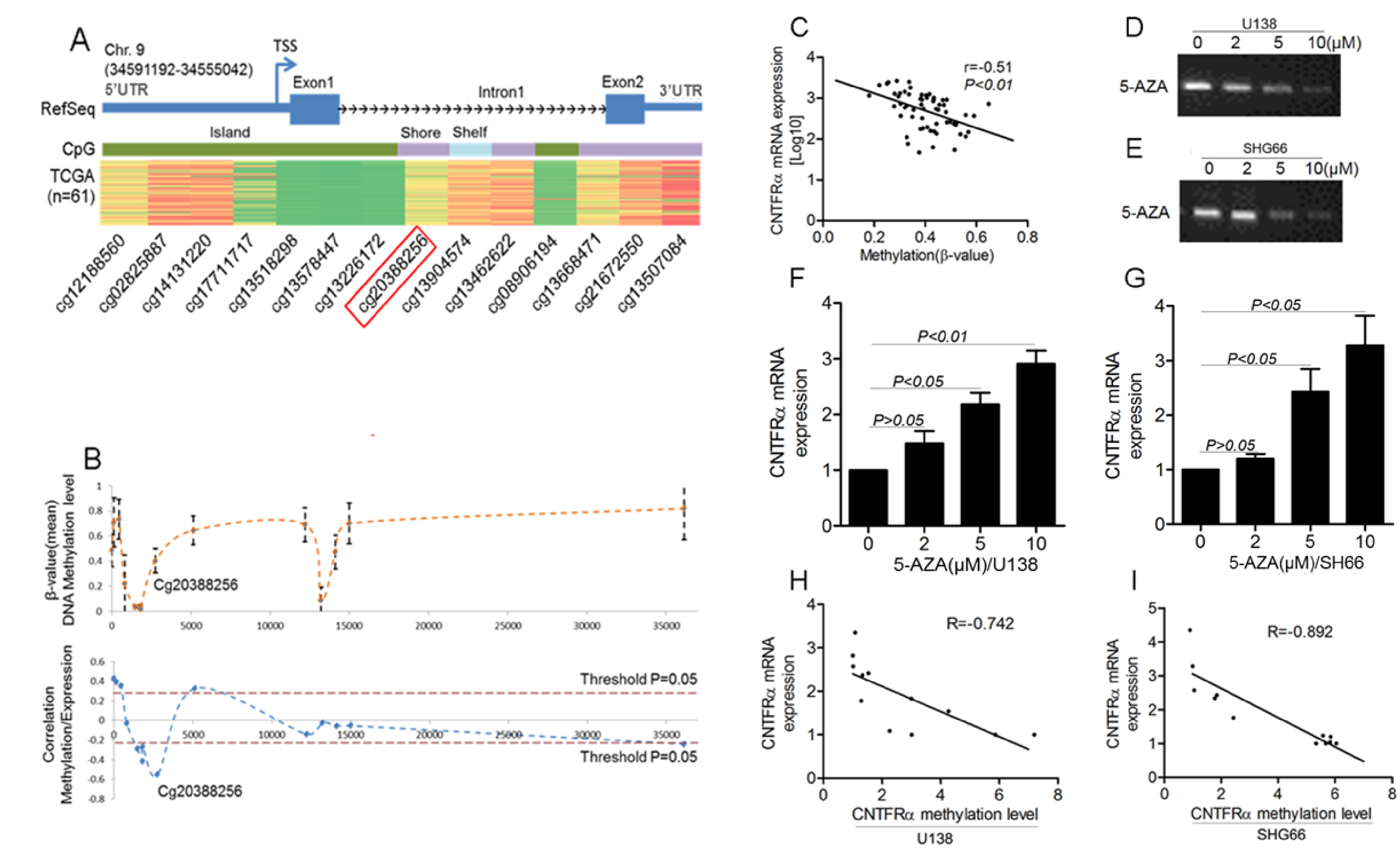

A
Chr. 9
(34591192-34555042)
TSS
5'UTR
Exon1
Intron1
Exon2
3'UTR
RefSeq
Island
Shore
Shelf
CpG
TCGA
(n=61)
cg12188560
cg02825887
cg14131220
cg17711717
cg13518298
cg13578447
cg13226172
cg20388256
cg13904574
cg13462622
cg08906194
cg13668471
cg21672550
cg13507084
B
β-value(mean)
DNA Methylation level
Cg20388256
Correlation
Methylation/Expression
Threshold P=0.05
Threshold P=0.05
Cg20388256
C
r=-0.51
P<0.01
CNTFRα mRNA expression
[Log10]
Methylation(β-value)
D
U138
0
2
5
10(μM)
5-AZA
E
SHG66
0
2
5
10(μM)
5-AZA
F
P<0.01
P<0.05
P>0.05
CNTFRα mRNA
expression
5-AZA(μM)/U138
G
P<0.05
P<0.05
P>0.05
CNTFRα mRNA
expression
5-AZA(μM)/SH66
H
R=-0.742
CNTFRα mRNA
expression
CNTFRα methylation level
U138
I
R=-0.892
CNTFRα mRNA
expression
CNTFRα methylation level
SHG66

Figure S3

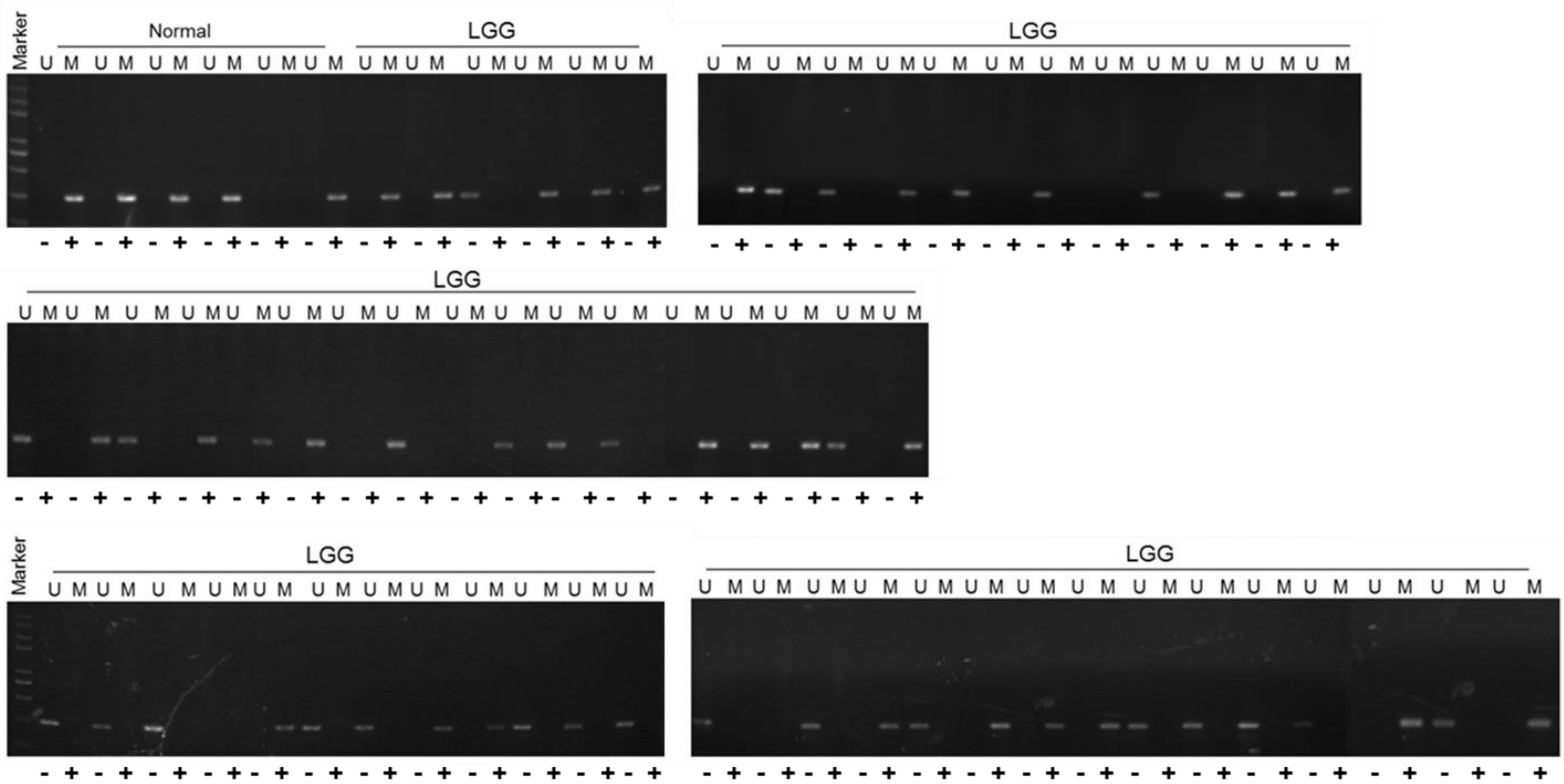

Figure S4

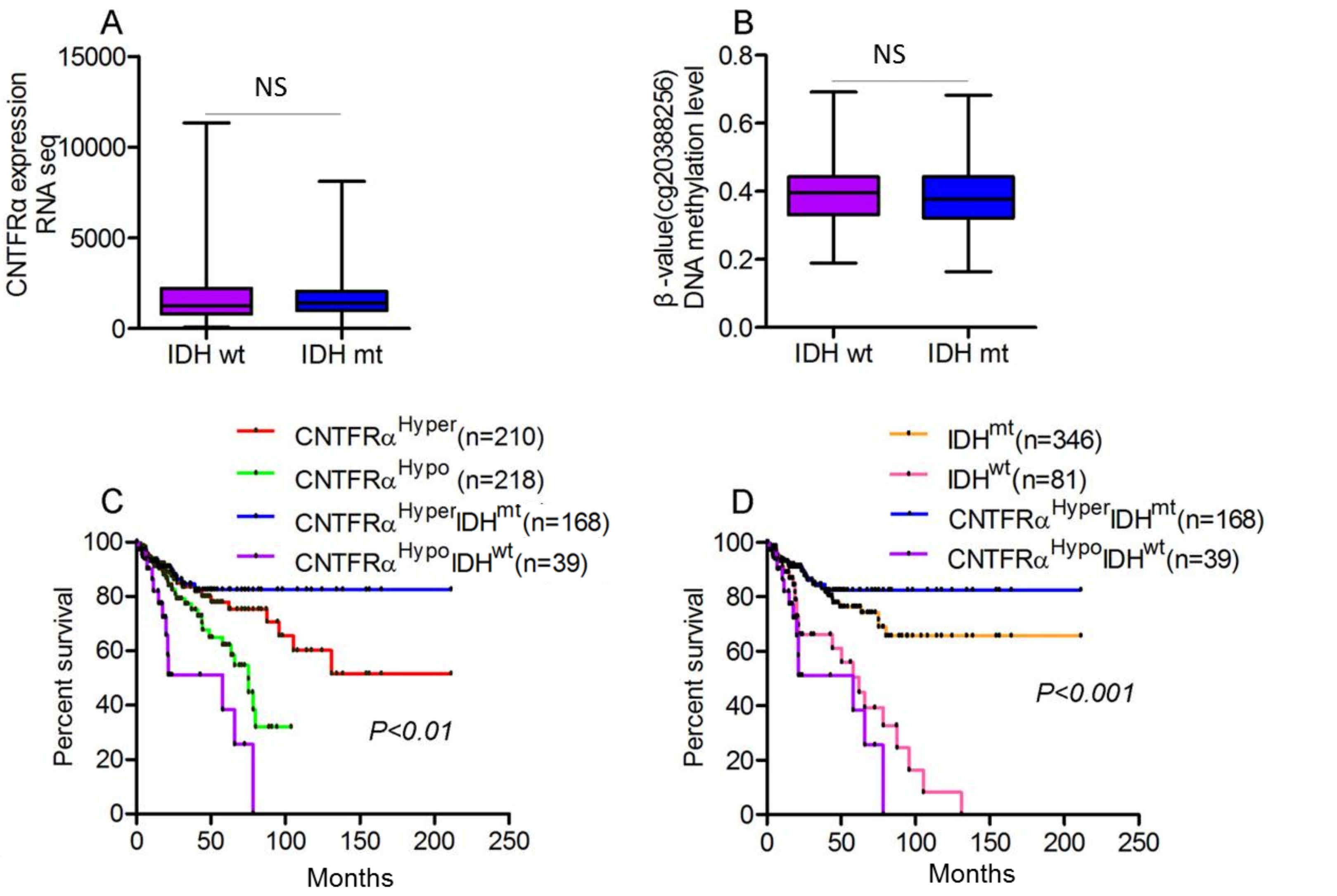
A
CNTFRα expression
RNA seq
15000
10000
5000
0
NS
IDH wt
IDH mt
B
β-value(cg20388256)
DNA methylation level
0.8
0.6
0.4
0.2
0.0
NS
IDH wt
IDH mt
C
CNTFRα Hyper (n=210)
CNTFRα Hypo (n=218)
CNTFRα Hyper IDH mt (n=168)
CNTFRα Hypo IDH wt (n=39)
Percent survival
100
80
60
40
20
0
0
50
100
150
200
250
Months
P<0.01
D
IDH mt (n=346)
IDH wt (n=81)
CNTFRα Hyper IDH mt (n=168)
CNTFRα Hypo IDH wt (n=39)
Percent survival
P<0.001
Months

Figure S5

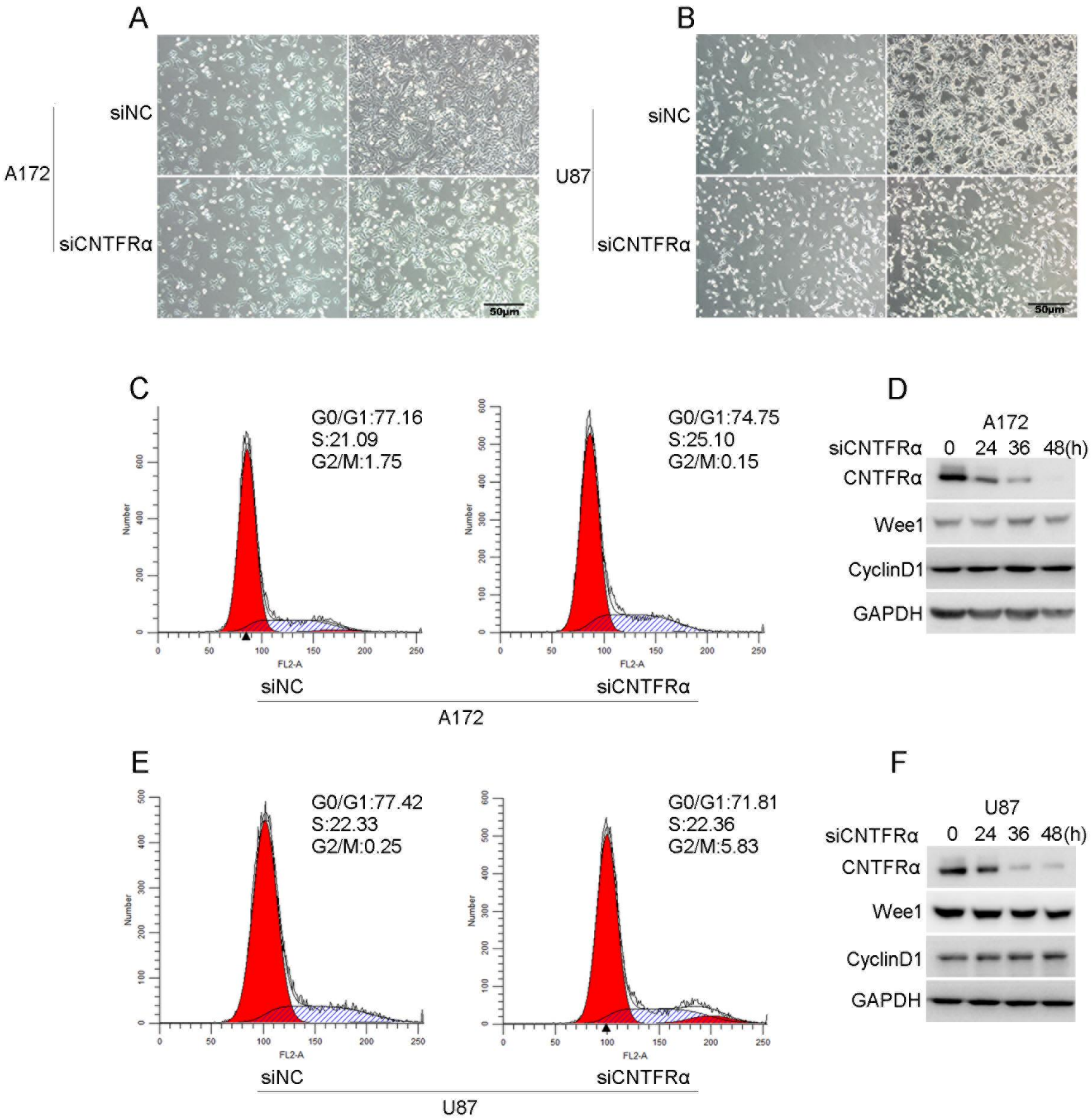

A
B
siNC
A172
siCNTFRα
50μm
siNC
U87
siCNTFRα
50μm
C
G0/G1:77.16
S:21.09
G2/M:1.75
G0/G1:74.75
S:25.10
G2/M:0.15
Number
FL2-A
siNC
siCNTFRα
A172
D
A172
siCNTFRα 0 24 36 48(h)
CNTFRα
Wee1
CyclinD1
GAPDH
E
G0/G1:77.42
S:22.33
G2/M:0.25
G0/G1:71.81
S:22.36
G2/M:5.83
siNC
siCNTFRα
U87
F
U87
siCNTFRα 0 24 36 48(h)
CNTFRα
Wee1
CyclinD1
GAPDH

Figure S6

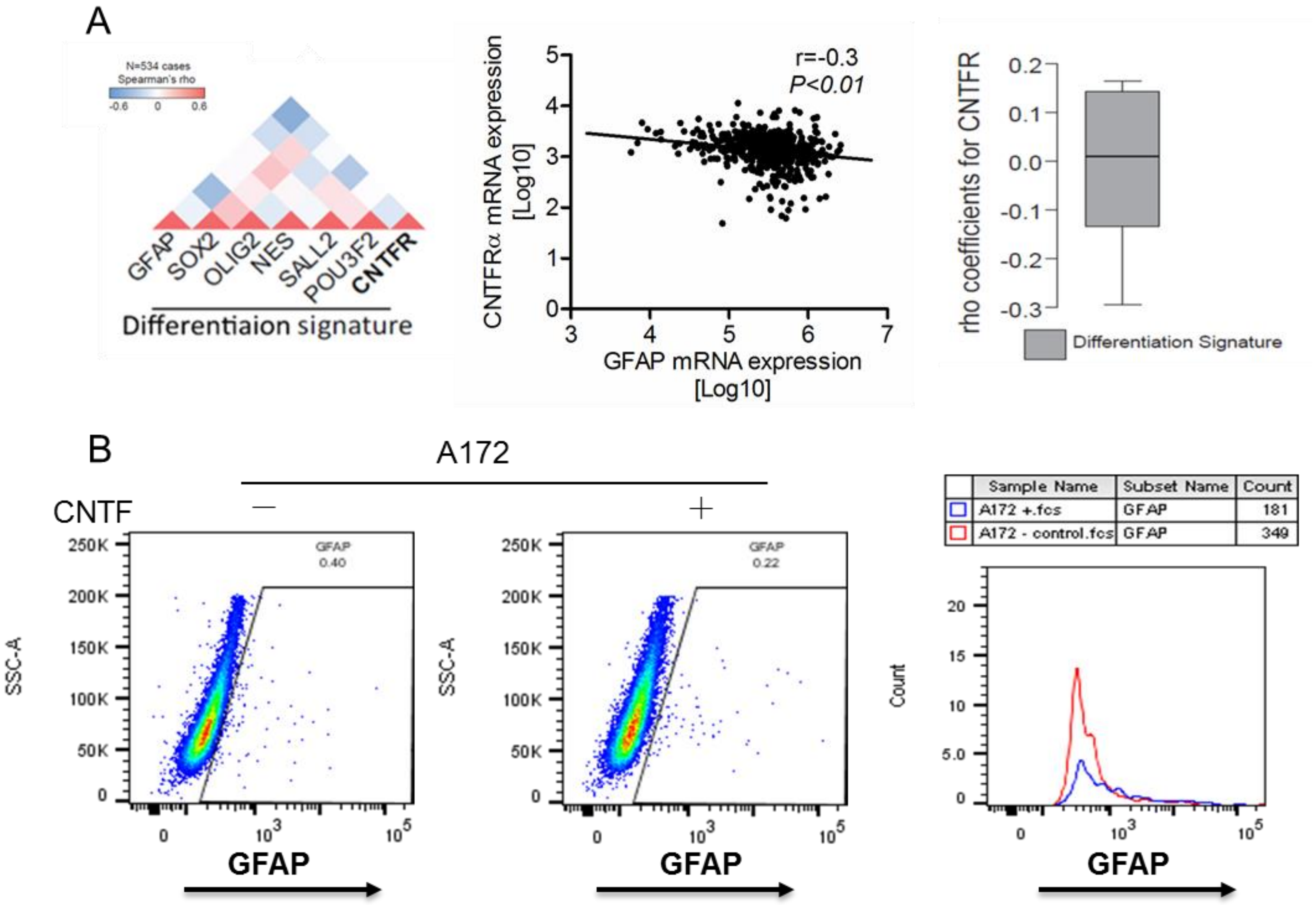

A
N=534 cases
Spearman's rho
-0.6
0
0.6
GFAP
SOX2
OLIG2
NES
SALL2
POU3F2
CNTFR
Differentiaion signature
r=-0.3
P<0.01
CNTFRα mRNA expression [Log10]
GFAP mRNA expression [Log10]
rho coefficients for CNTFR
Differentiation Signature
B
A172
CNTF
−
+
SSC-A
GFAP
0.40
GFAP
0.22
GFAP
Sample Name
Subset Name
Count
A172 +.fcs
GFAP
181
A172 - control.fcs
GFAP
349
Count
GFAP

Figure S7

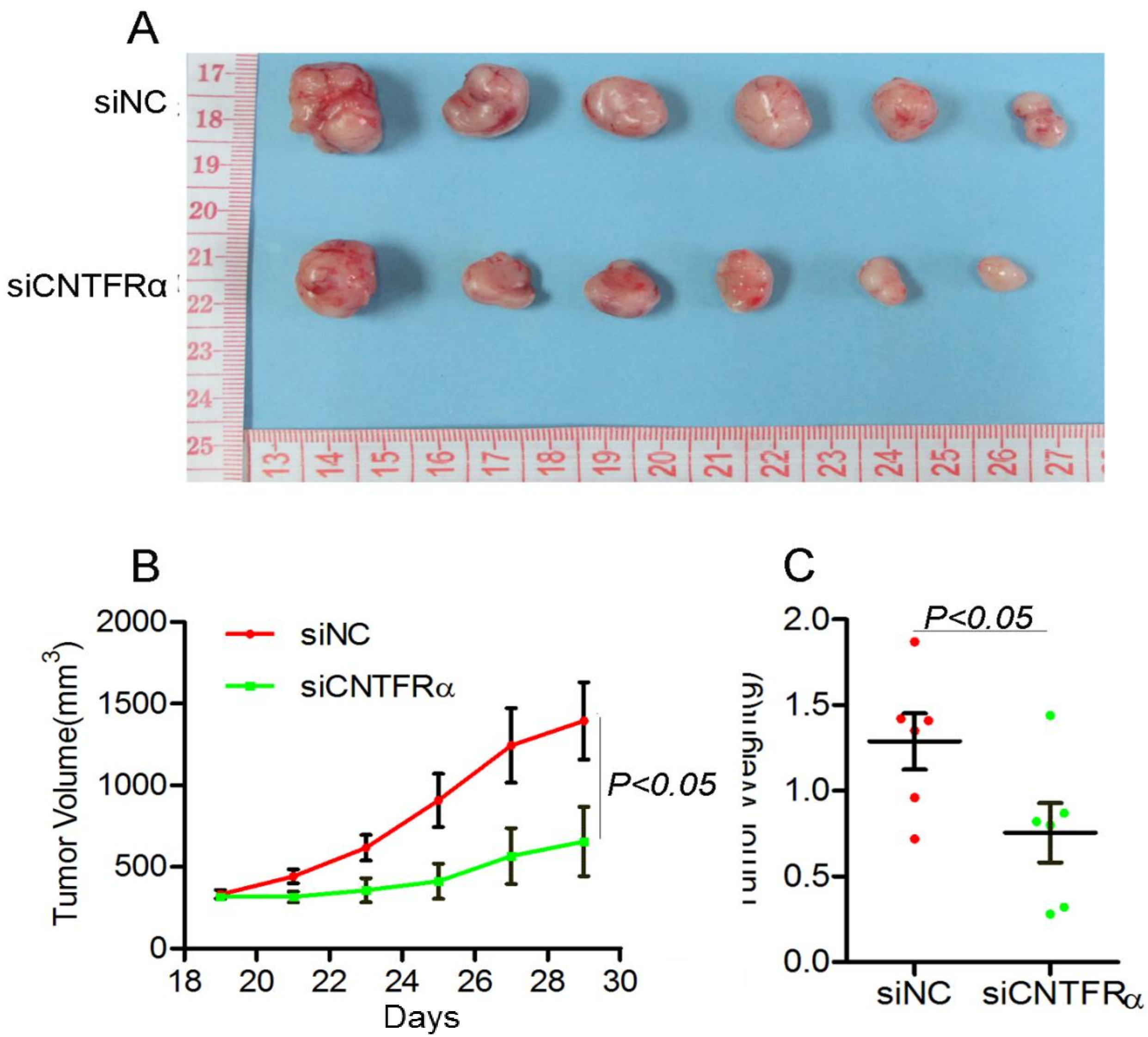

A
siNC
siCNTFRα
B
Tumor Volume(mm^3)
2000
1500
1000
500
0
18
20
22
24
26
28
30
Days
siNC
siCNTFRα
P<0.05
C
P<0.05
2.0
1.5
1.0
0.5
0.0
siNC
siCNTFRα
