# Supplementary material for: Hypomethylation of CNTFRα is associated with proliferation and poor prognosis in lower grade gliomas
Source: Sci Rep. 2017 Aug 1;7:7079. doi: 10.1038/s41598-017-07124-9 (PMC5539284; doi:10.1038/s41598-017-07124-9)
Supplement: Supplementary file 2 — Supplementary Figure legends [file 41598_2017_7124_MOESM2_ESM.pdf]

Hypomethylation of *CNTFRα* is associated with proliferation and poor prognosis in lower grade gliomas

Kun Fan[1,2†], Xiaowen Wang[1,3†], Jingwen Zhang[4†], Romela Irene Ramos[3], Haibo Zhang[1], Chunjie Li[1,2], Dan Ye[2], Jiansheng Kang[5], Diego M. Marzese[3], Dave S.B. Hoon[3,6], Wei Hua[1*]

Figure legends:

Fig. S1 mRNA expression of *CNTFRα* and *CNTF* in LGG and GBM. (A). Bioinformatic analysis of *CNTFRα* expression in LGG (n=530) and GBM (n=167, p<0.05, fold change = 0.98). (B). *CNTFRα* mRNA was more highly expressed in the proneural subtype compared with the other three subtypes of GBM in the TCGA cohort (n=202, p<0.001). (C). Bioinformatic analysis of *CNTF* expression in LGG (n=530) and GBM (n=167, p<0.05, fold change= 0.63).

Fig. S2 Methylation of CpG Island shore in *CNTFRα* regulated *CNTFRα* mRNA expression in GBM. (A) *CNTFRα* gene structure [based on RefSeq Feb. 2009 (GRCh37/hg19) assembly], and heat map showing DNA methylation level throughout the *CNTFRα* gene from the TCGA GBM cohort (n=61). Blue boxes represent the first and second exons. Green, purple and aqua represent the CpG context (CpG island,CpG shore and CpG shelf), respectively. The error bars represent the standard deviation (SD). (B) Correlation analysis between DNA methylation and gene expression levels. Each point represents one CpG site and the dashed line indicates the variation of correlation throughout *CNTFRα* gene. Orange lines represent the statistically significant threshold for the correlation analysis (p = 0.05). (C) Scatter plot of the correlation analysis between *CNTFRα* mRNA expression and methylation level of probe cg20388256 (Spearman correlation, r=-0.51, p<0.01). (D) and (E). Methylation status was evaluated in U138 and SHG66 by 5-Aza-2dC treatment, (F) and (G) mRNA of *CNTFRα* was increased by different doses of 5-Aza-2dC treatment, (H) and (I) *CNTFRα* mRNA expression had significant inverse correlation with CpG island shore methylation of *CNTFRα* Spearman correlation, r= -0.742 and -0.892, p<0.0001)

Fig. S3 MS-PCR detection of CNTFRα in LGG and normal brain tissues. LGG (n=62) and normal brain tissues (n=6) were detected by MS-PCR based on the probe cg20388256. Methylation status was defined as methylation or unmethylation.

Fig. S4 Combination of IDH mutation and CNTFRα methylation could have better survival assessment in LGG. Kaplan–Meier overall survival (OS) analysis according to methylation status of cg20388256 probe site and *IDH* mutant status. (A) *CNTFRα* mRNA was not significantly diffeent between *IDH* wt and mt (p>0.05). (B) Methylation status of cg20388256 probe was not significantly different between *IDH* wt and mt (p>0.05). (C) and (D) *CNTFRα*^{hyper}*IDH*^{mt} group had a better prognosis than the single methylation marker group (*CNTFRα*^{hyper} and *CNTFRα*^{hypo}), and *CNTFRα*^{hypo}*IDH*^{wt}group had a worse prognosis than the single mutation marker group (*IDH*^{wt} and *IDH*^{mt}). hyper: hypermethylation; hypo: hypomethylation; mt: mutation; mt: wide type.

Fig. S5 *CNTFRα* knockdown by targeting siRNA had no effect on the glioma cell cycle. (A) A172 and (B) U87 cell morphology was detected after siRNA transfection 48h. (C) and (E) Cell cycle was assayed by flow cytometry after siRNA transfection 48h in A172 and U87 cells. (D) and (F) Cell cycle proteins *Cyclin D1* and *Wee1* were detected by Western blot. Western blots represent three independent experiments.

Fig. S6 *CNTF* treatment reduced GFAP expression in glioma cells. (A) and (E) Correlation (Spearman rho) between *CNTF* and *CNTFRα* expression and differentiation gene signatures in 530 LGG patients. Red gradient: positive correlation. Blue gradient: negative correlation. (B). There was a significant inverse correlation between the *CNTFRα* and *GFAP* mRNA expression (Spearman correlation, r=-0.51, p<0.01). (C) and (D) A172 cells were treated with *CNTF* for 72 hours with low serum medium (1%), and then the *GFAP* was tested by flow cytometry. *GFAP* staining cells were significantly decreased after *CNTF* treatment.

Fig. S7 *CNTFRα* knockdown inhibited tumor xenograft growth *in vivo*. (A) U87 cells were injected subcutaneously into the flanks of nude mice. When xenograft tumor volume was even on the nineteen day, siRNA was injected into tumor xenograft every other day. Finally, tumor xenografts were harvested and photographed on the twenty-ninth day. (B) Tumor xenograft volume and (C) weight were calculated.
